# The Transcriptional Repressor Gfi1 Plays a Critical Role in the Development of NKT1- and NKT2-Type iNKT Cells

**DOI:** 10.1371/journal.pone.0157395

**Published:** 2016-06-10

**Authors:** Toshiaki Yasuoka, Makoto Kuwahara, Takeshi Yamada, Saho Maruyama, Junpei Suzuki, Masaru Taniguchi, Masaki Yasukawa, Masakatsu Yamashita

**Affiliations:** 1 Department of Obstetrics and Gynecology, Graduate School of Medicine, Ehime University, Shitsukawa, Toon, Ehime, Japan; 2 Department of Immunology, Graduate School of Medicine, Ehime University, Shitsukawa, Toon, Ehime, Japan; 3 Translational Research Center, Ehime University Hospital, Shitsukawa, Toon, Ehime, Japan; 4 Division of Immune Regulation, Department of Proteo-Inovation, Proteo-Science Center, Ehime University, Toon, Ehime, Japan; 5 Department of Infection and Host Defenses, Graduate School of Medicine, Ehime University, Shitsukawa, Toon, Ehime, Japan; 6 Department of Hematology, Clinical Immunology and Infectious Diseases, Graduate School of Medicine, Ehime University, Shitsukawa, Toon, Ehime, Japan; 7 Laboratory of Immune Regulation, RIKEN Center for Integrative Medical Sciences, 1-7-22 suehiro-cho, Tsurumi-ku, Yokohama, Japan; INEM, FRANCE

## Abstract

Gfi1 plays an important role in the development and maintenance of many hematopoietic linage cells. However, the impact of *Gfi1*-deficiency on the iNKT cell differentiation remains unclear. We herein demonstrate a critical role of Gfi1 in regulating the development of iNKT cell subsets. In the thymus of T cell-specific *Gfi1*-deficient mice, iNKT cells normally developed up to stage 2, while the number of stage 3 NK1.1^pos^ iNKT cells was significantly reduced. Furthermore, CD4^pos^ iNKT cells were selectively reduced in the peripheral organs of T cell-specific *Gfi1*-deficient mice. The α-GalCer-dependent production of IFN-γand Th2 cytokines, but not IL-17A, was severely reduced in T cell-specific *Gfi1*-deficient mice. In addition, a reduction of the α-GalCer-induced anti-tumor activity was observed in *Gfi1*-deficient mice. These findings demonstrate the important role of Gfi1 in regulating the development and function of NKT1- and NKT2-type iNKT cell subsets.

## Introduction

Invariant natural killer T (iNKT) cells are composed of a rare lymphocyte sub lineage with phenotypic and functional properties similar to T and NK T cells [1] [2]. Murine iNKT cells express the T cell antigen receptor (TCR) Vα14Jα18 chain coupled with Vβ8.2, Vβ7, or Vβ2, whereas human iNKT cells have Vα24Jα18 coupled to Vβ11. They play both effector and regulatory roles in infectious and autoimmune diseases. In cancer, they are mostly protective by producing IFN-γ to activate NK and CD8 T cells and by activating dendritic cells to release IL-12. iNKT cells have been recently shown to play a role in tumor immunosurveillance and the potential effect of these cells in tumor therapy is beginning to be uncovered. The ligand for iNKT cells was identified as α-galactosylceramide (α-GalCer), which is presented by CD1d and stimulates the immediate release of high levels of effector cytokines [1]. More recently, it was demonstrated that iNKT cells respond rapidly to a variety of glycolipids presented by CD1d and produce effector cytokines [3, 4]. In addition, the preferential production of Th2 cytokines from iNKT cells stimulated with OCH, a sphingosine-truncated α-GalCer, presented by CD1d has been reported [5].

iNKT cells mainly originate from CD4^+^CD8^+^ double positive (DP) progenitors in an agonist selection process involving endogenous ligands, including glycosphingolipid isoglobotrihexosyl ceramide in the thymus [6] [7] [8]. iNKT cell precursors recognize endogenous lipid antigens presented by CD1d, a homologue of the major histocompatibility complex (MHC). Importantly, they are selected by homotypic interactions of DP thymocytes that express CD1d on their cell surfaces. A subset of DP thymocytes express the transcription factor retinoic acid receptor-related Orphan Receptor γ(RORγt) and rearrange their Vα14 and Jα18 segments to form the invariant TCRαchain [9]. Following positive selection, iNKT cells downregulate CD8 and progress down a maturation pathway that is marked by the sequential acquisition of specific patterns of surface marker expression [10–12]. It is believed that maturation comprises four stages according to the expression of CD24, CD44 and NK1.1 molecules. The most immature stage of iNKT cells is defined as stage 0 (CD24^hi^CD44^lo^NK1.1^neg^) followed by stage 1 (CD24^lo^CD44^lo^NK1.1^neg^) and stage 2 (CD24^lo^CD44^hi^NK1.1^neg^). Mature iNKT cells are subdivided into at least 3 distinct populations, NKT1 (CD4^pos/neg^NK1.1^pos^), NKT2 (CD4^pos^NK1.1^neg^) and NKT17 (CD4^neg^NK1.1^neg^) [10–12]. NKT1 and NKT2 predominantly reside in non-lymphoid organs, while NKT17 cells mainly locate in the peripheral lymph nodes. NKT1 express T-bet and produce IFN-γand IL-4, whereas NKT2 cells express Gata3 and secret Th2 cytokines, IL-4 and IL-13. NKT17 cells produce Th17-related cytokines, IL-17 and IL-22.

Several transcriptional factors have been identified as regulators of iNKT cell development including Rorγt, Runx1, Heb, Egr2, NF-κB, Plzf, Gata3, T-bet, c-Myc and Id2 [10–12]. Among them, the broad complex Tramtrack bric-a-brac zinc finger (BTB-ZF) transcription factor Plzf is a key regulator of iNKT cell development and function [13, 14]. The expression of Plzf is largely restricted to iNKT cells, where it is highest in stage 0 and 1 populations. The *Plzf*-deficient mice showed an approximately 90% reduction in iNKT cell number and *Plzf*-deficient cells failed to acquire the characteristic features of iNKT cells in the thymus. However, the transgenic expression of *Plzf* in conventional T cells did not induce the expression of NKT cell-associated makers such as NK1.1, NKG2D, DX5 and 2B4, whereas the memory-like character was acquired [13, 15, 16]. Thus, the transcriptional regulation of iNKT cell development is not fully understood.

Gfi1 is a DNA binding transcriptional repressor, originally identified as a proto-oncogene that converts an IL-2-dependent cell line into an IL-2-independent cell line [17]. Gfi1 exerts its role as a transcriptional repressor by interacting with a number of histone modification enzymes including LSD1/CoRest, G9a and HDACs [18–21]. Gfi1 plays important roles in the differentiation of several hematopoietic cells including neutrophils, dendritic cells and B cells and in the maintenance of hematopoietic stem cells [22]. In CD4 T cells, it has been reported that Gfi1 regulates Th2 cell expansion via the enhancement of Stat5 activity [23, 24]. We previously reported that the expression level of Gata3 protein and the generation of IL-5-producing Th2 cells are severely impaired in *Gfi1*-deficient CD4 T cells [25]. Gfi1 acts as a downstream effector of the ERK/MAPK pathways and promotes the generation of IL-5-producing Th2 cells, in part by preventing Gata3 protein degradation. More recently, the Gfi1-mediated inhibition of Th17 and iTreg cells development has been reported [26–29]. Gfi1 negatively regulates *Il17a* expression, in part, via the inhibition of the recruitment of *Rorγt* to the *Il17a* promoter [26].

In this study, we showed that Gfi1 plays an important role in the development and/or maturation of iNKT cell subsets. CD4^pos^ and NK1.1^pos^ iNKT cell populations were significantly reduced in *Gfi1*^flox/flox^ CD4 Cre-transgenic (T cell-specific *Gfi1*-deficient: *Gfi1*-deficient) mice. The α-GalCer-dependent induction of IFN-*γ*, IL-4 and IL-13 production was severely reduced in *Gfi1*-deficient mice, whereas IL-17A was normally produced. Consistently, the decrease in the α-GalCer-induced anti-tumor activity was observed in *Gfi1*-deficient mice using a murine model of lung metastasis of B16 melanoma. These results clearly demonstrate that Gfi1 is a critical transcriptional regulator that controls the development of CD4^pos^ and NK1.1^pos^ iNKT cells.

## Materials and Methods

### Mice

Cre TG mice under the control of the *Cd4* promoter and Gfi1-EGFP knock-in mice were purchased from The Jackson Laboratory. *Gfi1*^flox/flox^ mice [30] were established and kindly provided by Dr. Jinfang Zhu (National Institute of Allergy and Infectious Diseases). C57BL/6 mice were purchased from Clea (Clea Japan, Inc., Tokyo, Japan). Both male and female mice were used in the *in vivo* and *in vitro* experiments. All mice were maintained under specific pathogen-free conditions and then used at 8–12 weeks of age. All of the animal experiments received approval from the Ehime University Administrative Panel for Animal Care. All animal care was conducted in accordance with the guidelines of Ehime University. All surgery was performed under anesthesia, and all efforts were made to minimize animal suffering and were used humane endpoints. Mice were monitored daily for deterioration in condition and signs of stress, as defined by lethargy, ruffled fur or a hunched appearance, at which time the mice were considered to have reached the ethically permitted humane endpoint criteria and were humanely euthanized using carbon dioxide asphyxiation.

### Reagents

α-galactosylceramide (α-GalCer) was purchased from Funakoshi (KRN7000).

The antibodies and CD1d tetramer used for cell-surface staining were as follows: α-GalCer-loaded APC-conjugated CD1d tetramer (cat#E001-4B; ProImmune), anti-NK1.1-PE (PK136; BD Biosciences), anti- CD4-FITC (RM4-5; BD Biosciences), anti-CD8-PE (53–6.7; BD Biosciences), anti-CD24-PE (M1/69; BioLegend), antip-CD24-APC (M1/69; BioLegend), anti-CD44-APC (IM7; BioLegend), anti-CD3εantibody-PE (145-2C11; eBioscience), anti-CD3εantibody-violetFluor 450 (17A2; TONBO Bioscience), anti-B220 antibody-PerCP/Cy5.5 (RA3-6B2; BioLegend), anti-IL17Rb-PE (MUNC33; eBioscience), and anti-CD19-PE (eBio1D2; eBioscience). All antibodies were diluted and used according to the manufacturer’s protocols.

A flow cytometric analysis (FACS) was performed using a Gallios flow cytometer (Beckman Coulter) or FACSCalibur cytometer (BD Biosciences), and the results were analyzed using the FlowJo software program (Tree Star).

### Intracellular staining of cytokines and transcription factors

Intracellular cytokine staining was then performed as described previously [31]. In case of an intracellular staining transcription factors, the cells were stained using a Transcription Factor Staining Buffer Kit according to the manufacturer’s protocol (cat#TNB-0607-KIT; TONBO biosciences). The antibodies used intracellular staining were as follows: anti-Rorγt-PE mAb (Q31-378; BD Biosciences), anti-Rorγt- Brilliant Violet 421 mAb (Q31-378; BD Biosciences), anti-T-bet-PE mAb (4B10; BioLegend), anti-T-bet-Brilliant Violet 421 mAb (4B10; BioLegend), anti-Gata3-PE mAb (L50-823; BD Biosciences), anti-Plzf-PE mAb (R17-809; BD Biosciences), anti-IFN-γ-FITC mAb (XMG1.2; BD Biosciences), anti-IFN-γ-PE mAb (XMG1.2; BD Biosciences), anti-IL-4-PE mAb (11B11; BD Biosciences), anti-IL-17A-PE mAb (TC11-18H10.1;BioLegend), or isotype controls (BD Biosciences).

### Enrichment of CD1d-tetramer^pos^ cells with magnetic cell sorter

The CD1d-tetramer^pos^ cells were enriched using a magnetic cell sorter as described previously [32]. Briefly, the thymocytes were stained with an α-GalCer-loaded APC-conjugated CD1d-tetramer, and the CD1d-tetramer^pos^ cells were then enriched using anti-APC microbeads (cat#130-090-855; Miltenyi Biotec) and an AutoMACs system.

### Isolation of iNKT cells by FACS sorting

The iNKT cells were purified by FACS sorting using a FACS Aria (BD Biosciences). The mononuclear cells of the indicated organs were stained with an α-GalCer-loaded CD1d-tetramer, anti-B220 mAb and anti-CD3ε. The α-GalCer-loaded CD1d-tetramer^pos^ B220^low^ CD3ε^pos^ cells were used as iNKT cells.

### Quantitative reverse transcriptase polymerase chain reaction

Total RNA was extracted from sorted iNKT cells. Total RNA was isolated using the TRIzol reagent and cDNA was synthesized using a Superscript VILO cDNA synthesis kit (cat#11754; Life Technologies). A quantitative RT-PCR analysis was performed as described previously, using a Step One Plus Real-Time PCR System (Life Technologies). The primer and TaqMan probe used for the detection of *Eomes* was purchased from Applied Biosystems. The expression of mRNA was normalized using the 18s rRNA signal. Specific primers, and Roche Universal Probes used in qRT-PCR were as follows: *18s* rRNA: 5’ GCAATTATTCCCCATGAACG 3’ (forward), 5’ GGGACTTAATCAACGCAAGC 3’ (reverse), probe #48; *Gfi1*: 5’ TCCGAGTTCGAGGACTTTTG 3’ (forward), 5’ GAGCGGACAGTGACTTCT 3’ (reverse), probe #7; *Tbx21*: 5’ TCAACCAGCACCAGACAGAG 3’ (forward), 5’ AAACATCCTGTAATGGCTTGTG 3’ (reverse), probe #19; *Plzf*: 5’ AATGCATTTACTGGCTCATTCA 3’ (forward), 5’ CAGGGCATCCTCCTTTGAG 3’ (reverse), probe #104; *Lef1*: 5’ TCCTGAAATCCCCACCTTCT 3’ (forward), 5’ TGGGATAAACAGGCTGACCT 3’ (reverse), probe #94; *Gata3*: 5’ TTATCAAGCCCAAGCGAAG 3’ (forward), 5’ TGGTGGTGGTCTGACAGTTC 3’ (reverse), probe #108; *Rorγt*: 5’ ACCTCTTTTCACGGGAGGA 3’ (forward) 5’ TCCCACATCTCCCACATTG 3’ (reverse), probe #6; *Zbtb7b*: 5’ CTTTGCCTGTGAGGTCTGC 3’ (forward), 5’ CAGTGGGGGCACGAGTAG 3’ (reverse), probe #2; *Runx1*: 5’ CTCCGTGCTACCCACTCACT 3’ (forward), 5’ ATGACGGTGACCAGAGTGC 3’ (reverse), probe #77; *Runx3*: 5’ TTCAACGACCTTCGATTCGT 3’ (forward), 5’ TTGGTGAACACGGTGATTGT 3’ (reverse), probe #103; *Il17rb*: 5’ GGACAGCCCTTCTTTGTCTG 3’ (forward), 5’ TGCTTTTTATATTTCATTACGTGGTT 3’ (reverse), probe #64; *Egr2*: 5’ CTACCCGGTGGAAGACCTC 3’ (forward), 5’ AATGTTGATCATGCCATCTCC 3’ (reverse), probe #60; *Ifnγ*: 5’ ATCTGGAGGAACTGGCAAAA 3’ (forward), 5’ TTCAAGACTTCAAAGAGTCTGAGGTA 3’ (reverse), probe #21; *Il4*: 5’ CATCGGCATTTTCAAGAG 3’ (forward), 5’ CGAGCTCACTCTCTGTGGT 3’ (reverse), probe #2; *Il13*: 5’ CCTCTGACCCTTAAGCAGCTTA 3’ (forward), 5’ CGTTGCACAGGGGAGTCT 3’ (reverse), probe #17; *Il17a*: 5’ CAGGGAGAGCTTCATCTGTGT 3’ (forward), 5’ GCTGAGCTTTGAGGGATGAT 3’ (reverse), probe #74.

### ELISA assay

The cells were stimulated with immobilized anti-TCR-βmAb (3 μg/ml) for 16 h. The concentrations of IFN-γand IL-4 in the supernatants were determined by an ELISA, as described previously [31]. For the determination of the IL-13 and IL-17A concentrations, the DuoSet ELISA Kit (cat#DY413 and DY421: R & D Systems) was used.

### Tumor lung metastasis model

B16 melanoma cells were maintained in complete DMEM (10% FBS, 50 U/ml penicillin, 50 μ/ml streptomycin). B16 melanoma (2×10^5^ cells/mouse) cells were intravenously injected into WT or *Gfi1*-deficient mice. A day after B16 melanoma injection, α-GalCer (4 μg/mouse) was intraperitoneally administered on days 5 and 9. The surface area of the lungs covered with B16 melanoma was determined using a Photoshop-based image analysis method. (Adobe Photoshop CS5 extended Version12.0 program) The data were recorded as the percentage of the mean pixel number of metastases.

### Statistical analysis

Student’s *t*-test was used for the statistical analyses. The survival rate was statically analyzed by the Kaplan-Meier actuarial methods, with statistical significance determined by the log-rank statistic using the SPSS statistical software program (SPSS Inc., Chicago, IL). A p value < 0.05 was considered to be statistically significant.

## Results

### CD4^pos^ iNKT cells are decreased in the peripheral organs of T cell-specific *Gfi1*-deficient mice

**Fig 1:**
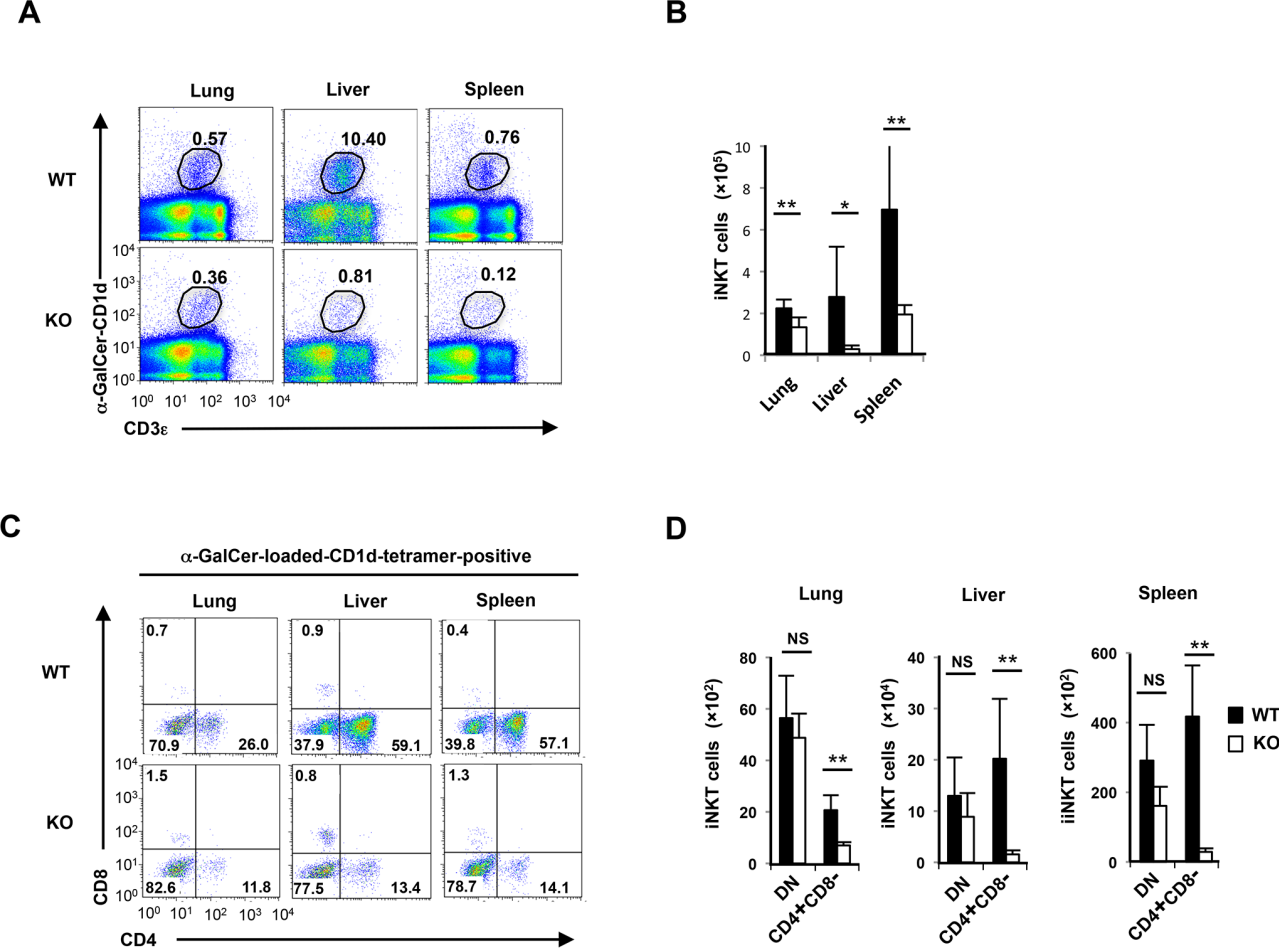
Decreased CD4^pos^ iNKT cell numbers in the peripheral organs of T cell-specific *Gfi1*-deficient mice. **(A)** The results of the flow cytometric analyses of iNKT cells from the lung, liver and spleen of T cell-specific *Gfi1*-deficient (*Gfi1* KO) mice. **(B)** The absolute number of iNKT cells in the lung, liver and spleen of wild-type (WT) and *Gfi1* KO mice (n = 4 for each group). **(C)** The results of the flow cytometric analyses of the CD4 and CD8 expression on WT and Gfi1 KO iNKT cells in the lung, liver and spleen. **(D)** The absolute number with the standard deviation of CD4^pos^ (CD4 SP) and CD4^neg^CD8^neg^ (DN) iNKT cells in the lung, liver and spleen of WT and *Gfi1* KO mice (n = 5 for each group). NS; not significant, *P<0.05, **P<0.01 (Student’s *t*-test).

**Fig 2:**
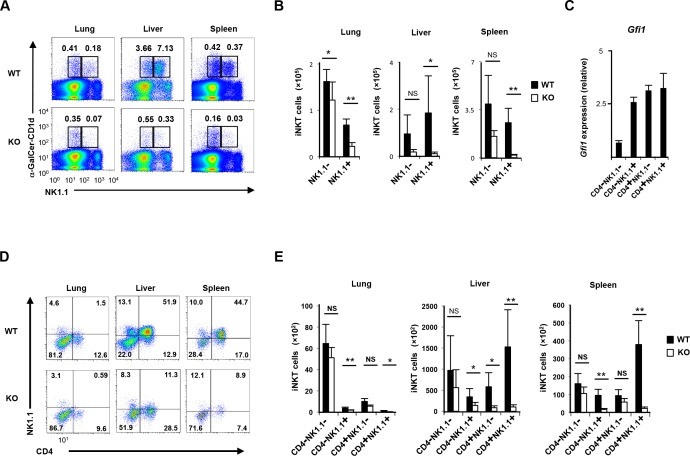
Decreased NK1.1^pos^ iNKT cell numbers in the peripheral organs of T cell-specific *Gfi1*-deficient mice. **(A)** The results of the flow cytometric analyses of the NK1.1 expression on the WT and *Gfi1* KO iNKT cells in the lung, liver and spleen. **(B)** The absolute number with the standard deviation of NK1.1^pos^ and NK1.1^neg^ iNKT cells in the lung, liver and spleen of WT and *Gfi1* KO mice (n = 5 for each group). **(C)** The results of the quantitative RT-PCR analysis of the *Gfi1* mRNA expression in the splenic iNKT cells. The splenic iNKT cells were divided into four populations according to the expression of CD4 and NK1.1. The results are presented relative to the mRNA expression of *18s* ribosomal RNA with the standard deviation (n = 3). **(D)** The results of the flow cytometric analyses of the CD4 and NK1.1 expression on WT and *Gfi1* KO iNKT cells in the lung, liver and spleen. **(E)** The absolute number with the standard deviation of CD4^neg^NK1.1^neg^, CD4^neg^NK1.1^pos^, CD4^pos^NK1.1^neg^ and CD4^pos^NK1.1^pos^ iNKT cells in the lung, liver and spleen of WT and *Gfi1* KO mice (n = 5 for each group). NS; not significant, *P<0.05, **P<0.01 (Student’s *t*-test).

It was previously reported that Gfi1 is expressed in thymocytes and mature peripheral T cells [22, 33]. We assessed the expression of Gfi1 in CD1d-restricted iNKT cells (iNKT cells) by a FACS analysis using heterozygote mutant mice expressing enhanced green fluorescent protein from the endogenous *Gfi1* locus (Gfi1-EGFP KI) [34]. Although the majority of iNKT cells in the spleen, lung and liver expressed EGFP, the expression level of EGFP was low in some NK1.1^neg^ iNKT cells in the spleen (**S1 Fig**). To determine the intrinsic role of Gfi1 in iNKT cells, we crossed *Gfi1*^flox/flox^ mice with *CD4-Cre* transgenic (TG) mice. The *Gfi1* gene was deleted in the CD4/CD8 DP stage in T cell thymic development. A significant decrease in the iNKT cell numbers in the spleen, liver and lungs was observed in T cell-specific *Gfi1*^flox/flox^ CD4-Cre (*Gfi1*-deficient) mice (**Fig 1A** and **1B**). The mature iNKT cells were then subdivided into two populations, CD4 iNKT cells and double CD4/CD8-negative (DN) iNKT cells [10, 11, 35]. We found that CD4^pos^ iNKT cells showed a striking reduction in the lung, liver and spleen of *Gfi1*-deficient mice, whereas DN iNKT cells remained unaffected (**Fig 1C** and **1D**). Furthermore, a selective reduction of the NK1.1^pos^ iNKT cell number was detected in the peripheral organs of *Gfi1*-deficient mice (**Fig 2A** and **2B**). The peripheral iNKT cells were subdivided into 4 populations according to the CD4 and NK1.1 expression [10, 11, 35]. Although a high-level expression of *Gfi1* mRNA was detected in the CD4^pos^NK1.1^pos^, CD4^pos^NK1.1^neg^ and CD4^neg^NK1.1^pos^ fractions of splenic iNKT cells, CD4^neg^NK1.1^neg^ iNKT cells expressed relatively lower levels of *Gfi1* mRNA (**Fig 2C**). A significant reduction in CD4^pos^NK1.1^pos^ and CD4^neg^NK1.1^pos^ iNKT cells was detected in the lung, liver and spleen of *Gfi1*-deficient mice (**Fig 2D** and **2E**). In addition, a decreased number of CD4^pos^NK1.1^neg^ iNKT cells was observed in the liver, but not in the lung and spleen of *Gfi1*-deficient mice (**Fig 2D** and **2E**). The number of CD4^neg^NK1.1^neg^ iNKT cells in the lung, liver and spleen was not affected by *Gfi1*-deficiency (**Fig 2E**).

### Gfi1 regulates NK1.1^pos^ iNKT cell development in the thymus

**Fig 3:**
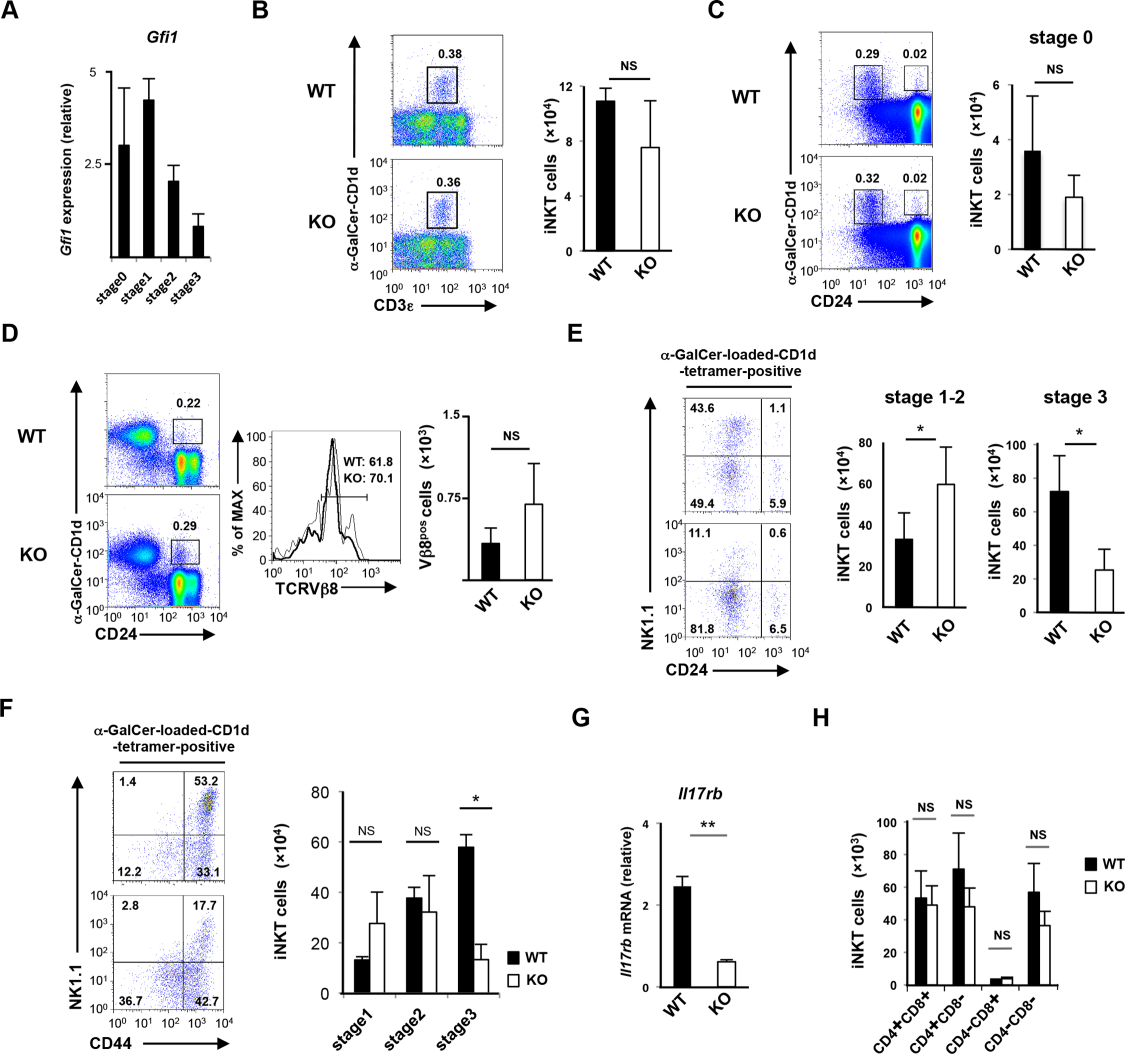
Gfi1 controls the transition from stage 2 to stage 3 during thymic iNKT cell development. **(A)** The results of the quantitative RT-PCR analysis of the *Gfi1* mRNA expression in thymic iNKT cells. The developmental stage of iNKT cells was defined by the expression of CD24, CD44 and NK1.1. The results are presented relative to the mRNA expression of *18s* ribosomal RNA with the standard deviation (n = 3). **(B, C, D** and **E)** The results of the flow cytometric analyses of thymic iNKT cells in *Gfi1* KO mice. A typical CD3εand α-GalCer-loaded CD1d tetramers pattern is shown **(B)**. The typical pattern of CD24 and α-GalCer-loaded CD1d tetramers (left) and the absolute numbers with the standard deviation of stage 0 (CD24^pos^) iNKT cells (right, n = 3) is indicated **(C)**. The CD1d-tetramer^pos^ thymocytes were enriched using a magnetic cell sorter and stained with anti-CD24 and anti-TCRVβ8 mAbs. The typical staining pattern of CD24 and α-GalCer-loaded CD1d tetramers (left), TCRVβ8 gated on CD24^high^, α-GalCer-loaded CD1d tetramer^pos^ cells (middle), and the absolute numbers with the standard deviation of Vβ8^pos^, CD24^high^, and α-GalCer-loaded CD1d tetramer^pos^ iNKT cells (right, n = 3) are indicated **(D)**. The typical pattern of CD24 and NK1.1 gated on α-GalCer-loaded CD1d tetramer-positive iNKT cells (left) and the absolute numbers with the standard deviation of stage 1–2 (CD24^low^NK1.1^neg^) iNKT cells and stage 3 (CD24^low^NK1.1^pos^) iNKT cells (right, n = 3) is indicated **(E)**. **(F)** The typical pattern of CD44 and NK1.1 gated on α-GalCer-loaded CD1d tetramer-positive iNKT cells (left) and the absolute numbers with the standard deviation of stage 1 (CD44^low^NK1.1^neg^), stage 2 (CD44^high^NK1.1^neg^) and stage 3 (CD44^high^NK1.1^pos^) iNKT cells (right, n = 3) is indicated. **(G)** The results of the quantitative RT-PCR analysis of the *Il17rb* mRNA expression in WT and *Gfi1*-deficient thymic iNKT cells. The results are presented relative to the mRNA expression of *18s* ribosomal RNA with the standard deviation (n = 3). **(H)** The absolute numbers of CD4^pos^CD8^pos^, CD4^pos^CD8^neg^, CD4^neg^CD8^pos^ and CD4^neg^CD8^neg^ iNKT cells in WT and *Gfi1* KO mice (n = 5 per group). NS; not significant, *P<0.05, **P<0.01 (Student’s *t*-test).

To further examine the role of Gfi1 on iNKT cell development, we next analyzed the development of iNKT cell in the thymus. Both immature (CD24^pos^) and mature (NK1.1^pos^) thymic iNKT fractions expressed considerable levels of EGFP (**S2 Fig**). The expression of *Gfi1* mRNA was also confirmed by a quantitative RT-PCR analysis. *Gfi1* mRNA was detected in iNKT cells of all developmental stages (stage 0 to stage 3), and the level was relatively higher in the earlier stages (stage 0 and stage 1) (**Fig 3A**). The *Gfi1*-deficient thymus had similar number of iNKT cells compared with the wild-type thymus (**Fig 3B**). Stage 0 CD24^high^ immature iNKT cells were not decreased in the *Gfi1*-deficient mice (**Fig 3C**). To further confirm the effect of Gfi1 deficiency on CD24^high^ immature iNKT cells, we assessed the Vβ8 bias of the TCR repertoire. The CD1d-tetramer^pos^ thymocytes were enriched using a magnetic cell sorter to avoid a nonspecific staining as described previously [32], and stained with an anti-CD24 and an anti-TCRVβ8 mAbs. The number of Vβ8^pos^ CD24^high^ immature iNKT cells in the thymus of *Gfi1*-deficient mice was comparable to that of in control WT mice (**Fig 3D**). In contrast, a decrease in stage 3 iNKT cells (CD24^low^NK1.1^pos^) and an increase in stage 1–2 iNKT cells (CD24^low^NK1.1^low^) were observed in the *Gfi1*-deficient mice (**Fig 3E**). The reduction of stage 3 iNKT cells and augmentation of the stage 1–2 iNKT cell ratio in the *Gfi1*-deficient thymocytes were confirmed by staining with NK1.1 and CD44 (**Fig 3F**). The expression of IL-17Rb is a maker for thymic iNKT cell subpopulations [36]. The expression of *Il17rb* mRNA in the *Gfi1*-deficient thymic iNKT cells was significantly reduced compared with the wild-type cells (**Fig 3G**). Surprisingly, the ratio of CD4 positive and negative iNKT cells was not altered by *Gfi1* deficiency in the thymus (**Fig 3H**). These data suggest that the reduction of CD4^pos^ iNKT cells occurs in the periphery, while the development of NK1.1^pos^ iNKT cells was altered in the thymus of *Gfi1*-deficient mice.

### Expression profile of transcriptional regulators in *Gfi1*-deficient iNKT cells

**Fig 4:**
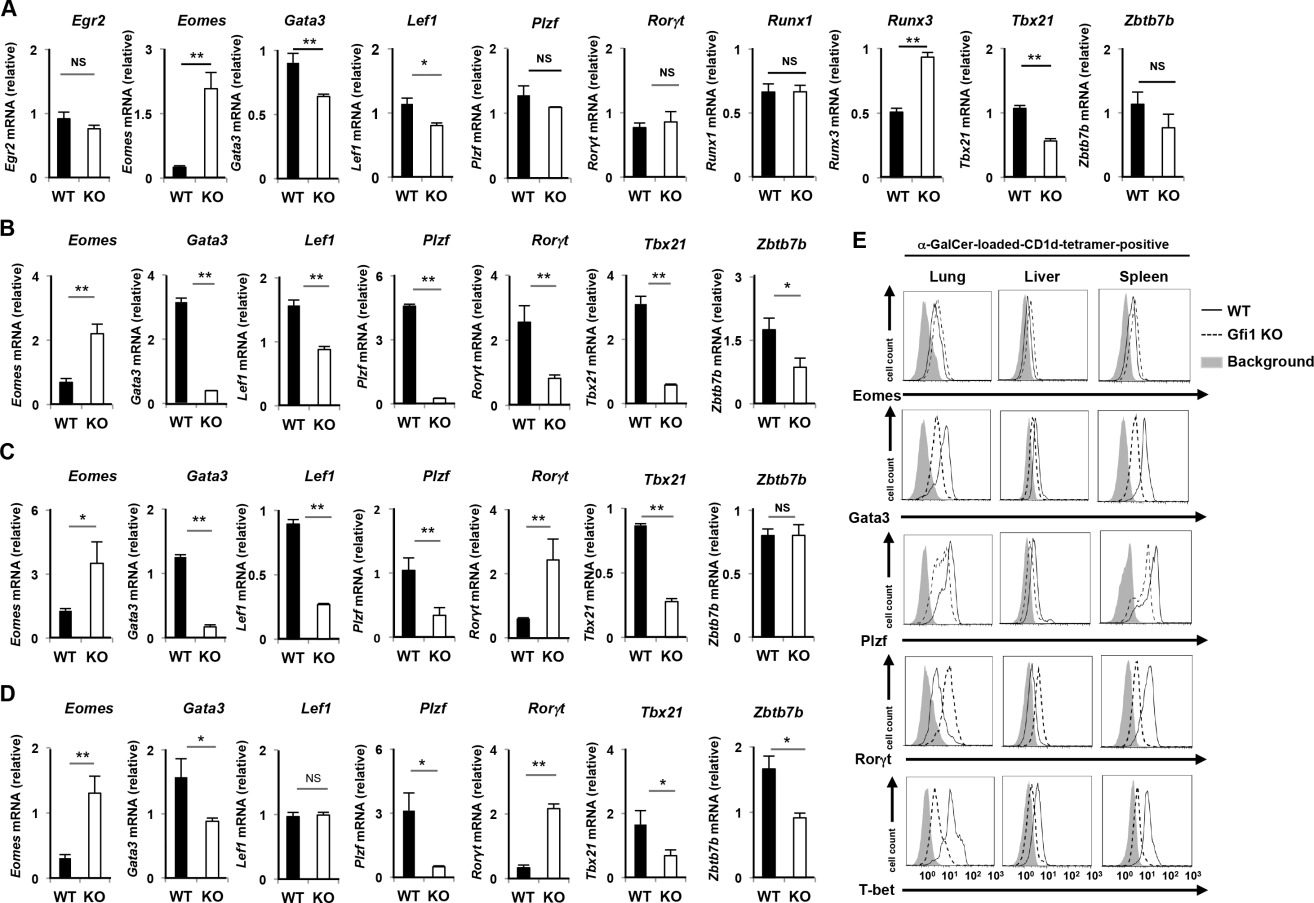
The expression profile of transcriptional regulators in *Gfi1*-deficient iNKT cells. The results of the quantitative RT-PCR analysis of transcriptional regulators in WT and *Gfi1*-deficient iNKT cells from the thymus **(A)**, spleen **(B)**, liver **(C)** and lung **(D)**. Each population was purified by FACS sorting. The results are presented relative to the mRNA expression of *18s* ribosomal RNA with the standard deviation (n = 3). **(E)** The results of the intracellular FACS analysis of Eomes, Gata3, Plzf, Rorγt and T-bet in iNKT cells from the indicated organs of WT and *Gfi1*-deficient mice. Three independent experiments were performed with similar results. NS; not significant, *P<0.05, **P<0.01 (Student’s *t*-test).

We next assessed the expression of transcriptional regulators, which play an important role in iNKT cell development and function. The α-GalCer-loaded CD1d-tetramer^pos^ B220^low^ CD3ε^pos^ mononuclear cells were isolated as total iNKT cells. The purity of the sorted iNKT (α-GalCer-loaded CD1d-tetramer^pos^ CD3ε^pos^) cells was greater than 99% (**S3A Fig**). We detected a dramatic increase in *Eomes* mRNA in the *Gfi1-*deficient thymic iNKT cells in comparison to the wild-type iNKT cells (**Fig 4A**). The expression of *Runx3* mRNA was also detected in *Gfi1*-deficient thymic iNKT cells (**Fig 4A**). Although the expression levels of *Gata3*, *Lef1* and *Tbx21* mRNA were significantly reduced in the *Gfi1*-deficient thymic iNKT cells compared with the wild-type cells, the differences were marginal (**Fig 4A**). *Plzf*, *Runx1*, *Zbtb7b*, *Egr2* and *Rorγt* mRNA were normally expressed in the thymic iNKT cells of *Gfi1*-deficient mice (**Fig 4A**). In contrast, splenic iNKT cells from *Gfi1*-deficient mice expressed lower levels of *Plzf*, *Gata3*, *Tbx21*, *Zbtb7b*, *Lef1* and *Rorγt*, and a higher level of *Eomes* mRNA compared with that of the wild-type cells (**Fig 4B**). The reduced levels of *Plzf*, *Gata3* and *Tbx21* and increased level of *Eomes* mRNA were also detected in the *Gfi1*-deficient iNKT cells from the liver and lung (**Fig 4C** and **4D**). However, *Rorγt* mRNA was increased in the *Gfi1*-deficient iNKT cells of the liver and lung, and the expression of *Zbtb7b* and *Lef1* showed different pattern in each of the organs (**Fig 4C** and **4D**). We next performed intracellular staining to assess the protein expression of the transcription factors. A decreased protein expression of Plzf, Gata3 and T-bet was detected in the *Gfi1*-deficient iNKT cells from the spleen, liver and lung (**Fig 4E**). The augmented expression of the Eomes protein in the *Gfi1*-deficient iNKT cells from these organs was also confirmed (**Fig 4E**). The expression of Rorγt was decreased in the *Gfi1*-deficient splenic iNKT cells, whereas the level was increased in the lung and liver (**Fig 4E**).

To understand the difference in the impact of *Gfi1*-deficiency on the expression of transcription factors in the iNKT cells between the organs, we divided splenic iNKT cells into 4 populations according to the CD4 and NK1.1 expression and examined the mRNA expression profile of the transcription factors. The mRNA expression of *Plzf*, *Gata3*, *Zbtb7b* and *Tbx21* was significantly low in the CD4^neg^NK1.1^neg^ iNKT cells (**S3B Fig**). In sharp contrast, the expressions of *Roγt* and *Eomes* mRNA were relatively higher in the CD4^neg^ iNKT cells compared with that of CD4^pos^ iNKT cells (**S3B Fig)**. These results support the notion that CD4- and NK1.1-positive iNKT cells were reduced in the peripheral organs of *Gfi1*-deficient mice.

**Fig 5:**
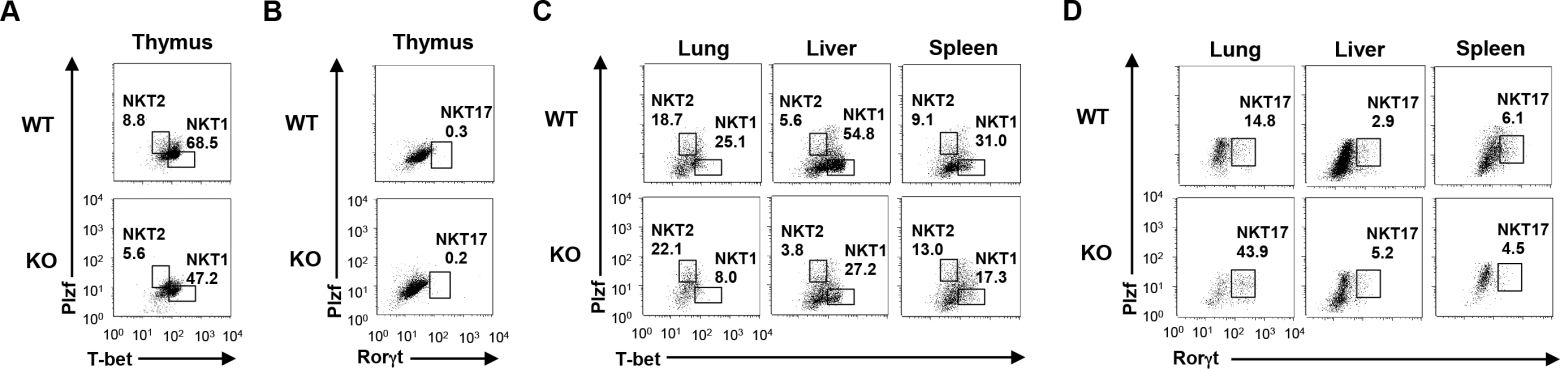
The development of iNKT1 (Plzf^low^T-bet^high^), iNKT2 (Plzf^high^T-bet^lowh^) and iNKT17 (Rorγt^high^) cells in *Gfi1*-deficient mice. iNKT cells were purified by FACS sorting and the intracellular staining of the indicated transcription factors was performed. The expression of Plzf and T-bet in the thymus **(A)** and the indicated peripheral organs **(C)**, and the patterns of Plzf and Rorγt in the thymus **(B)** and the indicated peripheral organs **(D)** are shown.

Finally, we assessed the difference of functional populations of iNKT cells between WT and *Gfi1*-deficient mice. The ratio of iNKT1 (Plzf^low^/T-bet^high^) and iNKT2 (Plzf^high^/T-bet^low^) cells was moderately reduced in the thymus of *Gfi1*-deficient mice, whereas the iNKT17 (Rorγt^high^) cells remained unaffected (**Fig 5A** and **5B**). The decreased ratio of iNKT1 cells was also detected in the lungs, liver and spleen of *Gfi1*-deficient mice (**Fig 5C**). The ratio of iNKT2 cells was moderately increased in the lung and spleen of *Gfi1*-deficient mice, but not in the liver (**Fig 5C**). The ratio of Rorγt-positive iNKT17 cells was increased in the lung and liver of the *Gfi1*-deficient mice (**Fig 5D**). Interestingly, the expression level of Rorγt in non-iNKT17 cells of the Gfi1-deficient mice was decreased (**Fig 5D**). These findings demonstrate the important role of Gfi1 in regulating the development and/or maintenance of type-1 iNKT cells.

### Decreased IFN-γand Th2 cytokine production of *Gfi1*-deficient iNKT cells

**Fig 6:**
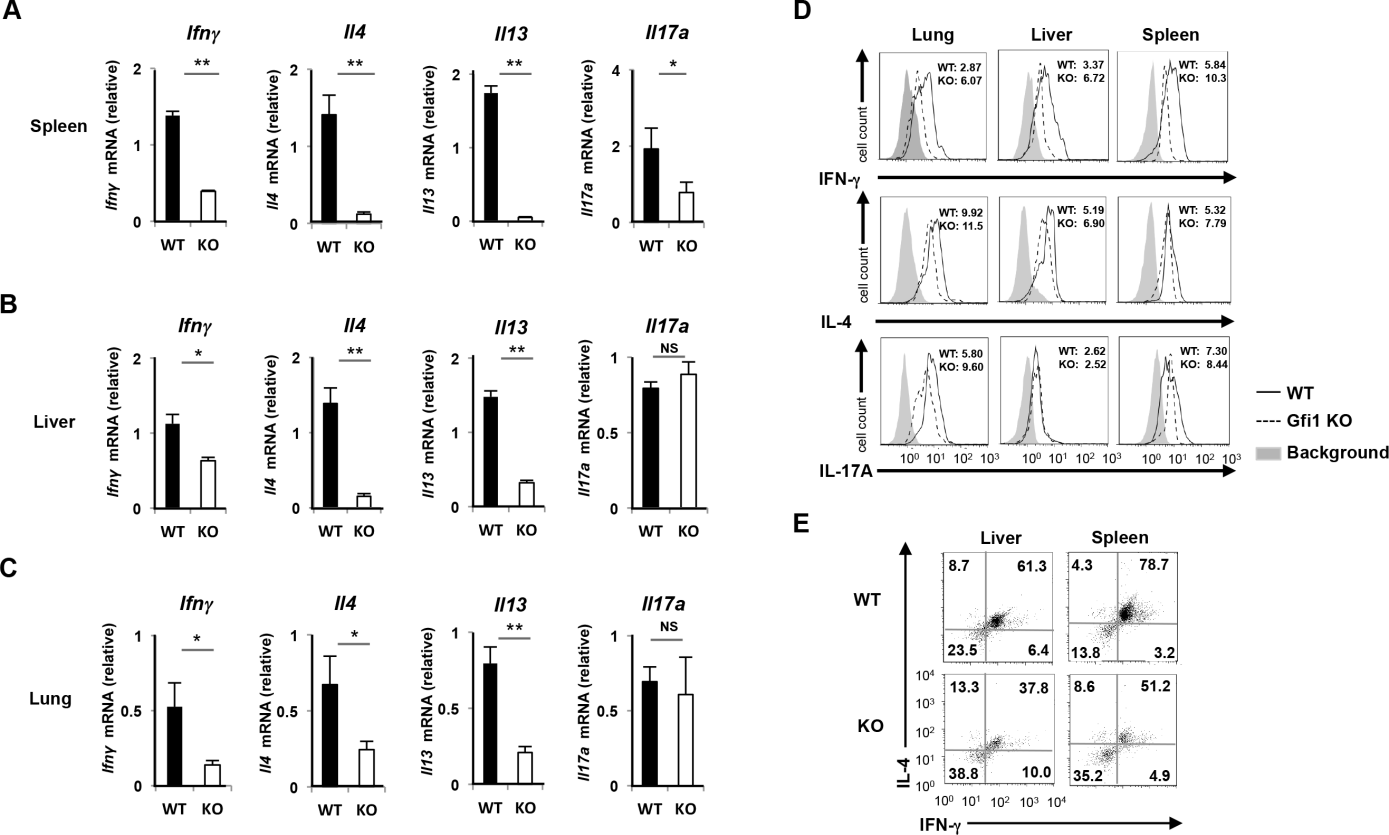
Reduction of IFN-γand Th2 cytokine production in *Gfi1*-deficient iNKT cells. The results of the quantitative RT-PCR analysis of cytokine mRNA in iNKT cells from the spleen **(A)**, liver **(B)** and lung **(C)** of WT and *Gfi1*-deficient mice. The iNKT cells were purified by FACS sorting and stimulated with PMA plus ionomycin for 2h. The results are presented relative to the mRNA expression of *18s* ribosomal RNA with the standard deviation (n = 3). NS; not significant, *P<0.05, **P<0.01 (Student’s *t*-test). **(D)** The results of the intracellular staining of the indicated cytokines in iNKT cells of the indicated organs. iNKT cells were purified by FACS sorting, and stimulated with PMA plus ionomycin in the presence of monensin for 4 h. The mean fluorescence intensities of the indicated cytokine stainings are shown. **(E)** The results of the intracellular staining of IL-4/IFN-γin the iNKT cells of the spleen and liver. iNKT cells were purified by FACS sorting, and stimulated with PMA plus ionomycin in the presence of monensin for 4 h.

We next examined the expression profile cytokines in splenic *Gfi1*-deficient iNKT cells. The α-GalCer-loaded CD1d-tetramer^pos^ B220^low^ CD3ε^pos^ iNKT cells were purified by FACS sorting (>98%) and then stimulated with PMA plus ionomycin for 2 h. The expressions of *Ifnγ*, *Il4* and *Il13* were severely attenuated in the *Gfi1*-deficient splenic iNKT cells compared with the wild-type splenic iNKT cells (**Fig 6A**). Similar results were obtained when we purified iNKT cells from the lung and liver (**Fig** 6**B** and 6**C**). In contrast, the reduced expression of *Il17a* mRNA was not detected in the *Gfi1*-deficient iNKT cells from the lung and liver (**Fig** 6**B** and 6**C**), and *Gfi1*-deficient splenic iNKT cells showed only a moderate reduction of *Il17a* mRNA (**Fig 6A**). The decreased IFN-γproduction in the *Gfi1*-deficient iNKT cells in the periphery was confirmed by intracellular staining (**Fig 6D, upper panel**). In contrast, there was a moderate reduction in the level of IL-4 proteins in the *Gfi1*-deficient peripheral iNKT cells (**Fig 6D, middle panel**). *Gfi1* deficiency only had a marginal effect on IL-17A production in iNKT cells (**Fig 6D, lower panel**). The number of IL-4/IFN-γdouble-producing splenic iNKT cells was also decreased in the spleen and liver of *Gfi1*-deficient mice (**Fig 6E**). Furthermore, we found that CD4^pos^NK1.1^pos^ iNKT cells stimulated with PMA plus ionomycin expressed high levels of *Ifnγ*and *Il4* mRNA, whereas the *Il17a* expression was predominantly induced in the NK1.1^neg^ iNKT cells (**S4 Fig**). Taken together, these results suggest that IFN-γ-, IL-4- and IL-13 producing iNKT cells are decreased in *Gfi1*-deficient mice via the selective reduction of CD4^pos^ and NK1.1^pos^ iNKT cells.

### α-GalCer-induced anti-tumor activity is attenuated by Gfi1-deficiency

**Fig 7:**
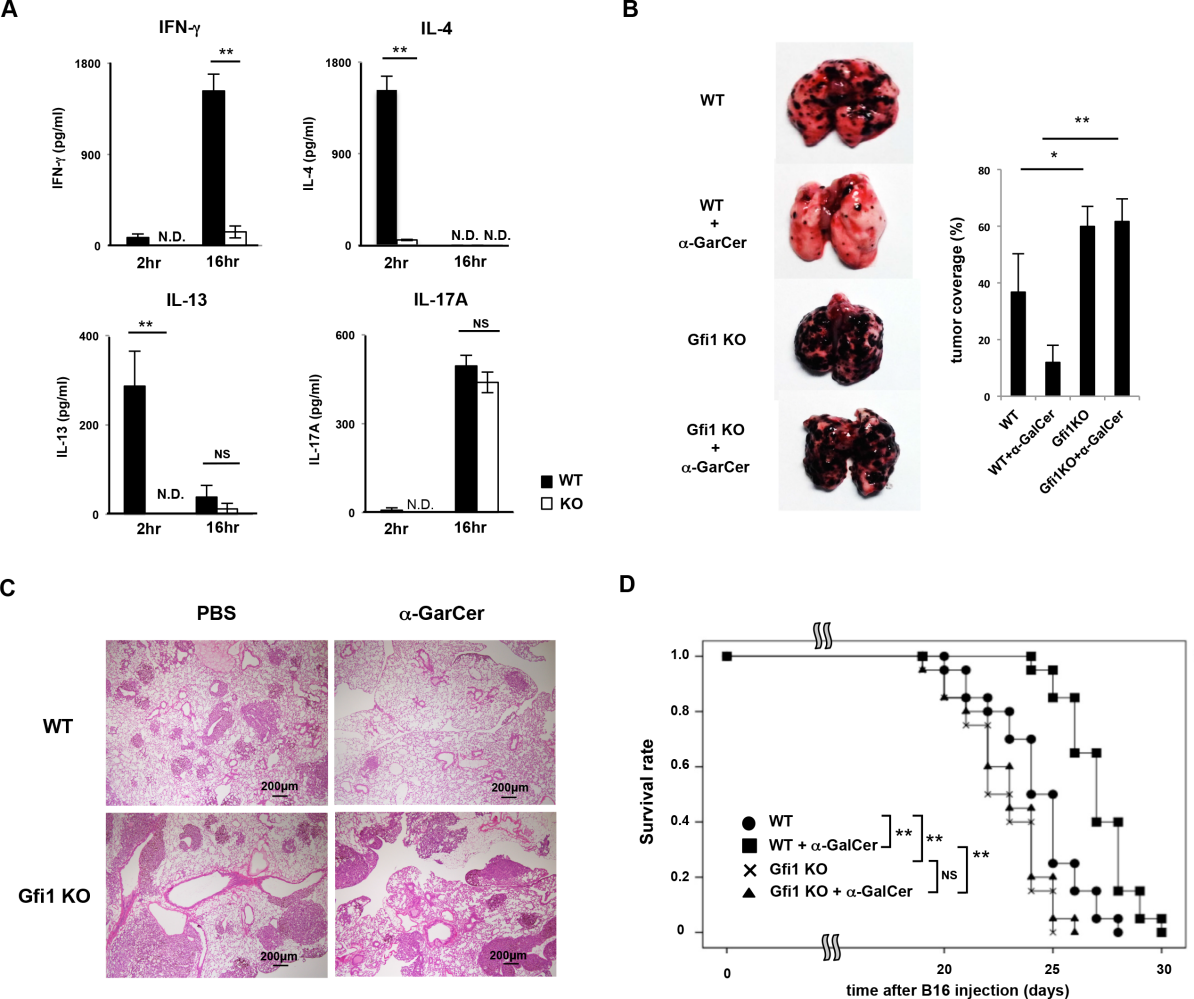
α-GalCer-induced anti-tumor activity is attenuated in *Gfi1*-deficient mice. **(A)** The amounts of cytokines in the serum at 2 and 16 h after the intravenous injection of α-GalCer (n = 5 for each group). N.D.; not detected, NS; not significant, *P<0.05, **P<0.01 (Student’s *t*-test). **(B)** Representative photographic views of metastasized lungs are shown (left). A quantitative analysis of tumor metastasis was performed by measuring the tumor coverage as described in the Materials and Methods. NS; not significant; *P<0.05, **P<0.01 (Student’s t-test). **(C)** Representative microscopic appearance of the lungs of wild-type and *Gfi1* KO mice fixed and stained with hematoxylin and eosin (H&E). Original magnification 40x (Scale bars = 200 μm). **(D)** The Kaplan-Meier curves representing the proportion of survival of mice in each group (n = 20 per group). P values were calculated with the log-rank (Mantel–Cox) test compared to the WT with α-GalCer administration group. **P<0.01.

Finally, we assessed the impact of the *Gfi1* deletion on iNKT cell-dependent immune response *in vivo*. To confirm the reduced production of IFN-γ, IL-4 and IL-13 in the *Gfi1*-deficient iNKT cells *in vivo*, α-GalCer was intravenously injected and the concentration of cytokines in the serum was measured at 2 and 16 h after the injection. As expected, the concentrations of IFN-γ, IL-4 and IL-13 were reduced in the α-GalCer–injected *Gfi1*-deficient mice compared with that of the wild-type mice (**Fig 7A**). The increase in the serum IL-17A level induced by α-GalCer injection was not affected by the *Gfi1*-deficiency (**Fig 7A**).

Next, we assessed the α-GalCer-dependent anti-tumor activity in *Gfi1*-deficient mice using the experimental lung metastasis model of B16 melanoma as previously reported [1]. An experimental schedule is indicated in **S5 Fig**. Briefly, B16 melanoma cells were intravenously transplanted on day 0, and α-GalCer was administrated intravenously on day 1, 5 and 9. Sixteen days after B16 melanoma injection, lung metastasis was determined. Lung metastasis of B16 melanoma cells assessed by tumor coverage was significantly suppressed by the α-GalCer administration in the wild-type mice (**Fig 7B** and **7C**). However, the administration of α-GalCer into *Gfi1*-deficient mice failed to inhibit lung metastasis of B16 melanoma cells (**Fig 7B**). Increased lung metastasis in the *Gfi1*-deficient mice was confirmed by hematoxylin and eosin (H&E) staining (**Fig 7C**). The survival time of the B16 melanoma-bearing wild-type mice was significantly extended by α-GalCer administration compared to non-treated wild-type mice (**Fig 7D**). The tumor-bearing *Gfi1*-deficient mice died more rapidly compared with the wild-type mice, and α-GalCer-dependent extension in the survival time was not observed (**Fig 7D**). These results indicate that α-GalCer/iNKT cell-dependent anti-tumor activity is impaired in the *Gfi1*-deficient mice.

## Discussion

In this study, we described the role of Gfi1 in the development and maintenance of both thymic and peripheral iNKT cells. Gfi1 drove terminal differentiation of thymic iNKT cells that required upregulation of the NK1.1 expression. In addition, Gfi1 maintained the CD4^pos^ iNKT cell number in the peripheral organs including the spleen, liver and lungs. Consequently, the production of IFN-γand Th2 cytokines, but not IL-17A, was significantly reduced in *Gfi1*-deficient iNKT cells both *in vitro* and *in vivo*. Furthermore, the α-GalCer-dependent anti-tumor activity was impaired in T cell-specific *Gfi1*-deficient mice. These results suggest that *Gfi1* ablation in DP thymocytes impacts on the development and maintenance of iNKT cells.

Mature iNKT cells are subdivided into at least 3 distinct populations, iNKT1 (CD4^pos/neg^NK1.1^pos^/Plzf^low^T-bet^high^), iNKT2 (CD4^pos^NK1.1^neg^/Plzf^high^T-bet^low^) and iNKT17 (CD4^neg^NK1.1^neg^/Rorγt^high^), according to the cytokine production ability [10, 11, 35]. iNKT1 cells express T-bet and produce IFN-γand IL-4, whereas iNKT2 cells express Gata3 and secret Th2 cytokines, IL-4 and IL-13. iNKT17 cells produce Th17-related cytokines, IL-17 and IL-22. We demonstrated that increase in the serum concentration of IFN-γ, IL-4 and IL-13 induced by α-GalCer intravenous injection were impaired in *Gfi1*-deficient mice. In contrast, the serum level of IL-17A in *Gfi1*-deficient mice was comparable to that in the wild-type mice. We sorted iNKT cells from the peripheral organs and stimulated them with PMA plus ionomycin to examine the cytokine expression in *Gfi1*-deficient peripheral iNKT cells. The reduced expression of *Ifnγ*, *Il4* and *Il13* mRNA was detected in *Gfi1*-deficient iNKT cells from the spleen, liver and lung. The intracellular staining of cytokines revealed that the number of IL-4/IFN-γdouble-producing cells was also decreased in the spleen and liver of *Gfi1*-deficient mice. The *Gfi1*-deficient iNKT cells from the peripheral organs showed a striking reduction of IFN-γexpression. Although the expression of *Il17a* was moderately decreased in the *Gfi1*-deficient splenic iNKT cells, iNKT cells from the liver and lung expressed a considerable amount of *Il17a* mRNA even in the absence of *Gfi1*. Furthermore, the decreased expression of T-bet and Gata3 was detected in the *Gfi1*-derficient iNKT cells from the peripheral organs. The reduced number of CD4^pos^ and NK1.1^pos^ mature iNKT cells was detected in *Gfi1*-deficient mice. Moreover, the number of Plzf^low^T-bet^high^ iNKT cells was decreased in *Gfi*1-deficient mice, whereas the Rorγt^high^ iNKT cells remained unaffected. Taken together, the *Gfi1*-deficiency has the strongest impact on the development of iNKT1 cells.

The expression of Rorγt was only reduced in the *Gfi1*-deficient splenic iNKT cells, and the increased level of Rorγt protein was detected in the *Gfi1*-deficient iNKT cells of the liver and lung. We cannot explain why the expression levels of Rorγt in iNKT cells were different in every organ of the *Gfi1*-deficient mice. We previously performed a DNA microarray analysis to compare the mRNA expression profiles of WT CD4 T cells and *Gfi1*-deficient CD4 T cells. The expression of *Ccr2*, *Ccr5* and *Ccr6* was significantly increased, whereas the level of *Ccr8* mRNA was reduced [37]. In addition, the increased expression of ItgαE was reported in *Gfi1*-deficient CD4 T cells [28]. Changes of the chemokine receptors and integrins in *Gfi1*-deficient iNKT cells would alter the organ distribution of the iNKT cell subsets. Thus, it is possible that the variations in the Rorγt level of the *Gfi1*-deficient mice were caused by the altered organ distribution of Rorγt-positive iNKT cells.

Gfi1 was originally identified as a proto-oncogene that prevents cell death of an IL-2-dependent cell line induced by IL-2 withdrawal [24]. IL-2 receptor (IL-2R) consists of an αchain, βchain and commonγ (γc) chain [38]. The γc chain is a cytokine receptor subunit that is common to the receptor complex for several different interleukin receptors including IL-4, IL-7, IL-9, IL-15 and IL-21 receptors. Furthermore, the IL-2Rβchain is also used as a component of IL-15 receptor. The critical role of IL-7 and IL-15 in iNKT cell development has been reported [39]. More recently, it was reported that Rorγt-positive NKT17 cells are IL-15 independent and instead rely completely on IL-7 [40]. We found that CD4^pos^ and NK1.1^pos^ iNKT cells were significantly reduced in T cell-specific *Gfi1*-deficient mice, whereas the number of CD4^neg^NK1.1^neg^ iNKT cells was less affected. It is likely that Gfi1 is induced by IL-15 and protects NKT1 (CD4^pos/neg^NK1.1^pos^) and NKT2 (CD4^pos^NK1.1^pos^) cells from cell death. In fact, the expression of Bcl-2 in stage 3 iNKT cells is significantly reduced in *Il15r*α-deficient mice and the number of NK1.1^pos^ thymic iNKT cell is decreased [38]. Moreover, it was previously reported that Gfi1 antagonizes p53-induced cell death by inhibiting the expression of pro-apoptotic factors including *Bax*, *Noxa* and *Puma* [41]. Gfi1 also protects hematopoietic stem cells against apoptosis [42]. The high-level expression of *Gfi1* mRNA was detected in the CD4^pos^ iNKT cells in the peripheral organs. Therefore, it is possible that Gfi1 supports the CD4^pos^ iNKT cells survival by inhibiting the expression of pro-apoptotic factors.

We found that stage 3 (NK1.1^pos^CD44^high^) thymic iNKT cells were significantly reduced in *Gfi1*-deficient mice, whereas stage 2 (NK1.1^neg^CD44^high^) iNKT cell numbers increased. The total thymic iNKT cell number was unaffected by *Gfi1*-deficiency. These results suggest that Gfi1 participates in the transition from stage 2 to stage 3 iNKT cells. T-bet plays an indispensable role in the final maturation of iNKT cells [43, 44]. The level of T-bet increases from stage 1 to 3 of thymic iNKT cell maturation, and iNKT cell development is blocked at stage 2 in the absence of T-bet. We found that the expression of *Tbx21* moderately decreased in *Gfi1*-deficient thymic iNKT cells compared with that in the wild-type cells, while another T-box family transcription factor, Eomes, was observed to increase. In addition, T-bet was preferentially expressed in CD4^pos^ iNKT cells in the spleen, whereas the expression of Eomes was predominant in CD4^neg^ iNKT cells. The increased level of Eomes may therefore contribute to the selective reduction of CD4^pos^ iNKT cells in T cell-specific *Gfi1*-deficient mice.

In this study, we used a CD4-Cre TG system to delete the *Gfi1* gene in T cells. Under these conditions, *Gfi1* is deleted after the CD4/CD8-double positive stage during thymic T cell development. Therefore, it is difficult to fully determine the intrinsic role of Gfi1 in iNKT cells. However, the data presented herein provide important information for improving our understanding of the development and maintenance of iNKT cells. We identified compelling evidence that Gfi1 mediates the development of iNKT cell subsets. Taken together, our present study thus provides new insight into iNKT cell development and maturation.

## Supporting Information

S1 Fig The results of the FACS analyses of heterozygous Gfi1-EGFP knockin mice. The expression of EGFP in iNKT cells of the lung, liver and spleen was analyzed by flow cytometry. (n = 3 per group).
(PDF)

S2 Fig The results of the FACS analyses of heterozygous Gfi1-EGFP knockin mice. The expression of EGFP in CD44-positive (left) and NK1.1-positive thymic iNKT cells (α-GalCer-loaded CD1d tetramer-positive cells) was analyzed by flow cytometry. (n = 3 per group).
(PDF)

S3 Fig **(A)** A representative staining profile of α-GalCer-loaded CD1d tetramer and CD3ε-stained purified iNKT cells. **(B)** The results of a quantitative RT-PCR analysis of transcriptional regulators in WT and *Gfi1*-deficient splenic iNKT cells. The splenic iNKT cells were divided into four populations according to the CD4 and NK1.1 expression. Each population was purified by FACS sorting and a quantitative RT-PCR analysis was performed. The results are presented relative to the mRNA expression of *18s* ribosomal RNA with the standard deviation (n = 3).
(PDF)

S4 Fig The results of the quantitative RT-PCR analysis of transcriptional regulators in WT and *Gfi1*-deficient splenic iNKT cells. The splenic iNKT cells were stained with CD4 and NK1.1 antibody and purified by FACS sorting. Then the cells were stimulated with PMA plus ionomycin for 2h, and a quantitative RT-PCR analysis was performed. The results are presented relative to the mRNA expression of *18s* ribosomal RNA with the standard deviation (n = 3).
(PDF)

S5 Fig A schematic representation of the experimental schedule for the lung metastasis of B16 melanoma. B16 melanoma (2×10^5^ cells/mouse) cells were intravenously inoculated on day 0, and α-GalCer was administered intravenously on days 1, 5 and 9. Sixteen days after B16 melanoma transplantation, lung metastasis was determined (n = 20 for each group).
(PDF)
