# Supplementary figures and images for: The Transcriptional Repressor Gfi1 Plays a Critical Role in the Development of NKT1- and NKT2-Type iNKT Cells
Source: PLoS One. 2016 Jun 10;11(6):e0157395. doi: 10.1371/journal.pone.0157395 (PMC4902269; doi:10.1371/journal.pone.0157395)

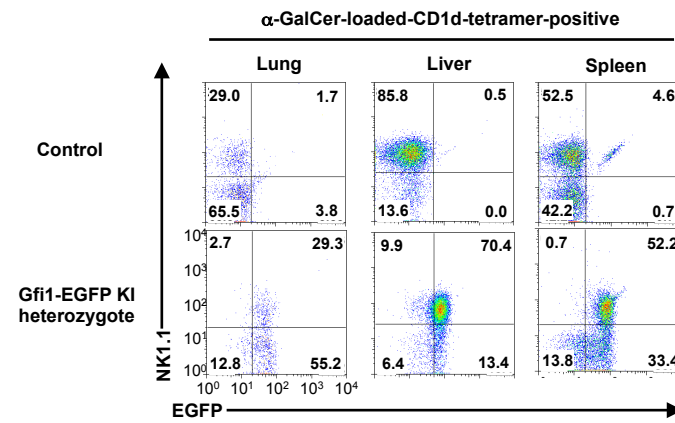

Supplement: S1 Fig — The expression of EGFP in iNKT cells of the lung, liver and spleen was analyzed by flow cytometry. (n = 3 per group). (PDF) [file pone.0157395.s001.pdf]

**$\alpha$ -GalCer-loaded-CD1d-tetramer-positive**

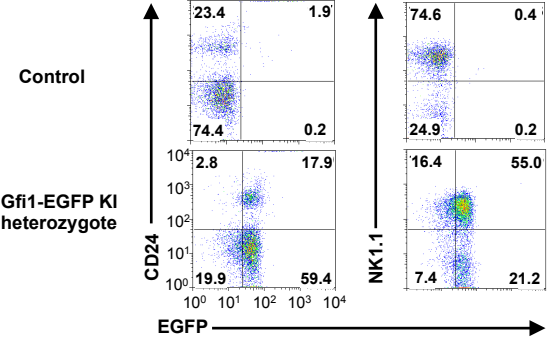

Supplement: S2 Fig — The expression of EGFP in CD44-positive (left) and NK1.1-positive thymic iNKT cells (α-GalCer-loaded CD1d tetramer-positive cells) was analyzed by flow cytometry. (n = 3 per group). (PDF) [file pone.0157395.s002.pdf]

**A**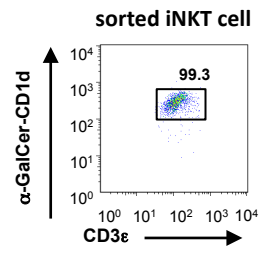**B**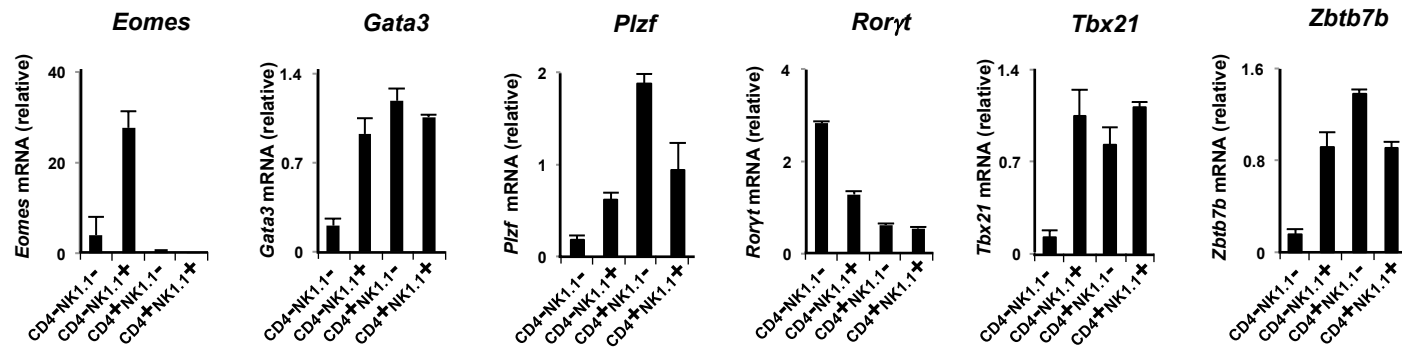

Supplement: S3 Fig — (A) A representative staining profile of α-GalCer-loaded CD1d tetramer and CD3ε-stained purified iNKT cells. (B) The results of a quantitative RT-PCR analysis of transcriptional regulators in WT and Gfi1-deficient splenic iNKT cells. The splenic iNKT cells were divided into four populations according to the CD4 and NK1.1 expression. Each population was purified by FACS sorting and a quantitative RT-PCR analysis was performed. The results are presented relative to the mRNA expression of 18s ribosomal RNA with the standard deviation (n = 3). (PDF) [file pone.0157395.s003.pdf]

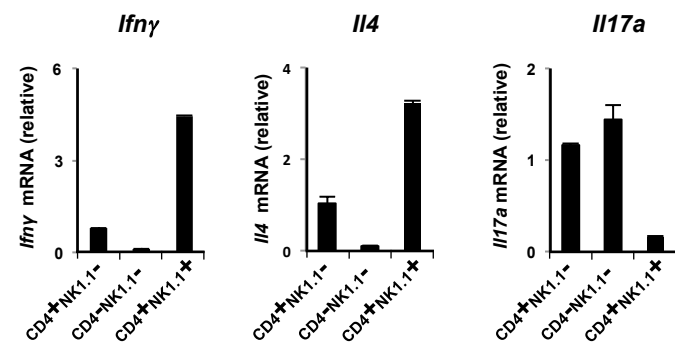

Supplement: S4 Fig — The splenic iNKT cells were stained with CD4 and NK1.1 antibody and purified by FACS sorting. Then the cells were stimulated with PMA plus ionomycin for 2h, and a quantitative RT-PCR analysis was performed. The results are presented relative to the mRNA expression of 18s ribosomal RNA with the standard deviation (n = 3). (PDF) [file pone.0157395.s004.pdf]

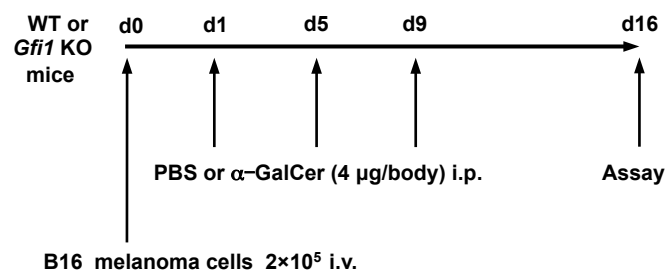

Supplement: S5 Fig — B16 melanoma (2×10^5^ cells/mouse) cells were intravenously inoculated on day 0, and α-GalCer was administered intravenously on days 1, 5 and 9. Sixteen days after B16 melanoma transplantation, lung metastasis was determined (n = 20 for each group). (PDF) [file pone.0157395.s005.pdf]
